# Novel Online or Mobile Methods to Assess Eating Patterns

**DOI:** 10.1007/s13668-017-0211-0

**Published:** 2017-07-11

**Authors:** Felicity J. Pendergast, Rebecca M. Leech, Sarah A. McNaughton

**Affiliations:** Institute for Physical Activity and Nutrition (IPAN), School of Exercise and Nutrition Sciences, Deakin University, Geelong, 221 Burwood Highway, Burwood, Victoria 3125 Australia

**Keywords:** Dietary assessment, Eating occasion, Food diary, Food record, Technology, Systematic review

## Abstract

**Purpose of Review:**

Recent developments in technology-based dietary assessment allow real-time data collection of eating occasions, yet their application to assessing eating pattern constructs has not been evaluated. The purpose of this review was to examine existing electronic and mobile food diary methods in relation to their ability to assess eating patterns constructs (e.g. patterning, format and context of eating occasions).

**Recent Findings:**

A systematic search of electronic databases identified 18 dietary assessment methods. Multiple methods with diverse technological capabilities have been developed, yet few studies report on their ability to assess all eating pattern constructs, particularly eating occasion context. Validity of the methods to assess overall dietary intake was found to be similar to traditional dietary assessment methods.

**Summary:**

A diverse range of methods are available for examining the patterning and format/content, but not context, of eating occasions. Further consideration of eating pattern constructs is required when developing dietary assessment methods.

## Introduction

High-quality dietary assessment underpins all areas of research and practice in the field of nutrition and dietetics. Dietary assessment is used to evaluate the types and amounts of foods and beverages consumed [1] and may be used to assess a variety of exposures including nutrients, foods, eating occasions [2] and overall diet through assessment of diet quality and dietary patterns [3]. Traditionally, nutritional epidemiology has focused on assessing the relationship between nutrient or food intakes and specific health outcomes. Recently, there has been increasing interest in examining eating patterns [4••, 5•]. The study of eating patterns is important as humans do not consume individual nutrients or foods in isolation; instead, they consume a varied diet with foods and beverages, usually consumed together at eating occasions such as meals or snacks [2]. The concept of eating patterns encompasses three key domains or constructs of interest (1) patterning of eating occasions (for example, frequency, spacing, regularity, skipping and timing), (2) eating occasion format or content (for example, food combinations, nutrient content and sequencing of foods) and (3) context (for example, eating with others, location of eating and activity whilst eating) [5•, 6]. Increasing research suggests that the timing and distribution of food intake or distribution of eating occasions across the day, not just the total amount of nutrients or foods, may be important for health and well-being [7] and that eating context may influence eating behaviours and dietary intake [5•].

While there is an established need to examine eating patterns [8], there are still major research gaps [4••, 9]. Research examining eating occasions and eating pattern constructs at the population level has been limited due to a number of methodological challenges associated with dietary assessment. Existing methods rarely allow assessment of eating patterning, format and context [10]. Many existing studies rely on the use of single questions or short questionnaires to assess eating patterns, and these measures have unknown validity [11] and cannot provide assessments of timing or content of eating occasions [10]. Similarly, food frequency questionnaires, where participants report their frequency of consumption of a specified list of foods, do not provide data on individual eating occasions or timing of food intake across the day. Only 24-h recall methods (where the respondent is asked to recall all food and beverage intake during the previous day) or food diaries or records (a record of all food and beverages eaten over a set period of time) can provide the necessary data to examine eating occasions. However, 24-h recall methods depend on episodic memory processes [12]. Due to their prospective data collection methods, food diaries offer the most promise for assessing eating occasions and the associated eating pattern constructs of pattering, format and context, although current food diary methods have high participant and researcher burden.

Due to the inherent complexities in assessing what people eat, the field of dietary assessment has looked to technology to assist in advancing current food diary methods. New technologies using electronic and mobile methods such as computers, handheld personal digital assistants (PDA) and mobiles phones have the potential to overcome many of the limitations associated with traditional pen and paper food diary methods [13••]. They can allow real-time data collection to study food consumption in the settings in which the food is consumed and allow the study of microprocesses that influence eating patterns in real-world contexts [14]. Real-time data collection involves the prospective and repeated sampling of a person’s behaviour and experiences within their natural environment, a process known as ecological momentary assessment (EMA) [15]. Electronic and mobile methods have the potential to reduce participant burden and improve compliance associated with the more detailed measures of food intake but also improve data quality by reducing measurement error and bias [14, 16, 17]. They may also reduce researcher burden by decreasing costs and resources associated with data collection, coding and reporting [14].

To date, applying technology in dietary assessment has primarily focused on introducing improvements relating to data entry and mode of administration (e.g. mobile and web-based tools) [18], improvements relating to coding and analysing food intake [19] and augmentation of data collection (e.g. use of wearable devices/cameras) [20–22]. While existing reviews of electronic or mobile methods have focused on technology aspects [14], their application to the study of eating pattern constructs has not been evaluated. This study aimed to conduct a systematic review of the existing electronic and mobile food diary methods in relation to their ability to assess eating patterns constructs (e.g. patterning, format and context of eating occasions).

## Methods

### Search Strategy

Online databases (Academic Search complete, CINAHL Complete, PsycINFO, SocINDEX, Applied Science and Technology and Business Source Complete) were searched through EBSCO Host. MEDLINE Complete, Global Health, Scopus, EMBASE and Web of Science for peer-reviewed original human research studies published in English between January 1994 and March 16th 2017. Bibliographies of included articles were also reviewed (hand searched) for additional articles. Search terms were tested prior to the recorded search to ensure that appropriate articles were identified. The following search terms were used: ((food* OR diet* OR nutrient*) N3 (consum* OR habit* OR intak* OR measur*)) OR eat* OR pattern* OR occasion* OR environment* OR context* AND (assess* OR method* OR monitor* OR analy* OR evaluat* OR valid*) AND (“Information communication*” OR technology OR “personal digital assistant*” OR PDA OR computer OR internet OR “information science”* OR “radio waves”* OR “radio frequency” OR photo* OR digital OR “smart phone*” OR “mobile phone*” OR “cell phone*” OR blackberry* OR image* OR camera* OR electronic* OR application* OR Wii OR app OR apps AND (food* OR diet*) N2 (record* OR diary*).

### Eligibility Criteria

For an article to be included in this review it was required to meet the following criteria: (1) original research article, published in a peer-reviewed journal, with full text in English language; (2) dietary assessment was conducted on human participants; (3) method of dietary assessment was classified as a ‘food record’ or ‘food diary’; (4) food diary or record utilised an element of technology; (5) dietary assessment took place in a free-living setting; and (6) the study design reported the evaluation or validation of the food record against a known reference method.

Articles were excluded if they met any of the following criteria: (1) studies published as abstracts, conference proceedings, poster or not in the English language; (2) dietary assessment was conducted on animals other than humans; (3) method of dietary assessment reported was not a ‘food diary’ or ‘record’; (4) the food diary was not exclusively completed on a technologic platform, e.g. smartphone or computer; (5) dietary assessment was conducted outside of a free-living setting, e.g. school cafeteria, residential care facility or laboratory; (6) study design was descriptive or did not include an evaluation/validation in comparison to an established dietary reference method and/or a biomarker of dietary intake (e.g. urine nitrogen, plasma carotenoids) and/or a direct measure of energy expenditure (e.g. doubly labelled water (DLW) method, pattern-recognition activity monitors or physical activity diaries); and (7) the dietary assessment tool did not assess total dietary intake.

### Study Selection

The titles and abstracts were independently reviewed by two reviewers (FJP, RML). Articles that did not meet eligibility criteria were excluded, and the remaining full-text articles were screened for inclusion. For discrepancies between reviewers about article eligibility, a third reviewer (SAM) was consulted.

### Data Extraction and Synthesis

Data extraction was conducted by two independent reviewers (FJP, RML) using an electronic spreadsheet, with the extraction verified by the alternate reviewer and discrepancies confirmed with the third reviewer (SAM). Information extracted included author, name of dietary assessment tool, platform and device, population group, country, features (data entry input, EMA prompts, GPS capabilities and feedback to participants), coding method and finally eating pattern assessment (patterning, format and context). The data extracted regarding the evaluation/validation of each dietary assessment method included reference method and time frame, dietary intake variables and statistical results. These headings were based on previous reviews and research examining eating pattern assessment and technology-based dietary assessment [5•, 14, 23].

## Results

**Fig. 1:**
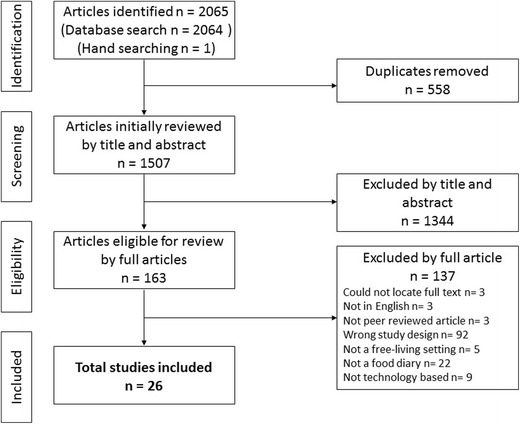
Flow diagram of included articles

Of the 2065 articles identified, 1507 were screened based on their title and abstract (Fig. 1). Of these, 163 full-text articles were assessed for eligibility and 26 studies were included in the review [24–49]. From these 26 studies, 18 separate dietary assessment methods were reported. Each published paper was treated as a separate study throughout this review, given they were reported using different reference methods or were an updated version of the test method. Table 1 presents details of the food diary dietary assessment methods in the included studies. Table 2 presents the details of the evaluation/validation of each dietary assessment method.

**Table 1:** Characteristics of existing online and mobile food diaries available used for assessing dietary intake and the applicability to assessing pattern variables

First author (ref), year	Name of dietary assessment method/tool	Platform and device	Population	Country	Features				Coding method	Eating Pattern Assessment		
					Data Entry Input	EMA prompts	GPS capability	Feedback to participants		Patterning	Format	Context
Kaczkowski [33], 2000	Multimedia Diet Record (MMDR)	Microcassette, tape recorder and camera	Adults; *N* = 53; 100% female; mean (SD) age = 64.9 (11.3) years	Canada	I, V	No	No	No	MCD	No	Yes	No
Wang [48], 2002	Wellnavi method	App, PDA	Young adults; *N* = 20; 100% female; age NR (University students)	Japan	I, T	No	No	No	MCD	No	Yes	No
Beasley [26], 2005	Diet Mate Pro	App, Smartphone	Adults; *N* = 39; 54% female; mean (SD) age = 53 (1.7) years	US	FD	No	No	No	AFD	Yes	Yes	No
Wang [47], 2006	Wellnavi method	App, PDA	Young adults; *N* = 28; 100% female; mean (SD) age = 19.3 (0.5) years	Japan	I, T	No	No	No	MCD	No	Yes	No
Kikunaga [34], 2007	Wellnavi method	App, PDA	Adults; *N* = 75; 64% female; mean (SD) age = 48.8 (10.2) years	Japan	I, T	No	No	No	MCD	No	Yes	No
Beasley [25], 2008	Diet Mate Pro	App, Smartphone	Adults (BMI > 25 kg/m^2^); *N* = 89; 83% female; mean (SD) age = 52 (12) years	US	FD	Yes	No	No	AFD	Yes	Yes	No
Fukuo [29], 2009	PDA-based food diary	App, PDA	Young adults; *N* = 44 without diabetes; 55% female; mean (SD) age = 23.2 (2.5) years; *N* = 16 with diabetes; 19% female; mean (SD) age = 52.8 (9.9) years	Japan	FD	No	No	No	AFD	Yes	Yes	No
Higgins [30], 2009	Photographic FR	Camera	Children; *N* = 28; 50% female; mean (SD) age = 12.6 (2.0) years	US	I	No	No	No	MCD	No	Yes	No
McClung [37], 2009	BalanceLog	App, PDA	Young adults; *N* = 13; <1% female; mean (SD) age = 23 (4) years	US	FD	No	No	No	AFD	No	Yes	No
Rollo [42], 2011	Nutricam Dietary Assessment Method (NuDAM)	App, Smartphone	Adults (type 2 diabetes); *N* = 10; 40% female; mean (SD) age = 61.2 (6.9) years	AUS	I, V	Yes (In follow up phone call)	No	No	MCD	Yes	Yes	Yes
Touvier [46], 2011	NutriNet-Sante Web-based 24-h dietary record	Internet-based program, Computer	Adults; *N* = 147; 59.2% female; mean (SD) age = 60.8 (6.0) years	France	FD, T	Yes	No	No	AFD	Yes	Yes	Yes
Martin [36], 2012	Remote Food Photography Method (RFPM)	App, Smartphone	Adults (BMI > 25 kg/m^2^); *N* = 40; 77.5% female; mean (SD) age = 40.3 (14.3) years	US	I	Yes	No	No	SAIA	No	Yes	No
Carter [27], 2013	My Meal Mate (MMM)	App, Smartphone	Adults; *N* = 50; 72% female; mean (SD) age = 35 (9) years	UK	I, FD	No	No	No	MCD	Yes	Yes	No
Hutchesson [32], 2013	Online FR	Internet-based program, Computer	Adults; *N* = 9; 100% female; mean (SD) age = 34.5 (11.3) years	AUS	FD	No	No	Yes	AFD	No	Yes	No
Astell [24], 2014	Novel Assessment of Nutrition and Ageing (NANA)	Internet-based program, Computer fitted with Webcam	Older adults (BMI > 25 kg/m^2^); *N* = 40; 60% female; mean (SD) age = 72.39 years	UK	FD, I, V	No	No	No	MCD	Yes	Yes	No
Hutchesson [31], 2015	Online FR	App, Smartphone OR Internet-based program, Computer	Young adults; *N* = 18; 100% female; mean (SD) age = 23.4 (2.9) years	AUS	FD	No	No	Yes	AFD	No	Yes	No
Monnerie [49], 2015	Estimated online FR	Internet-based program, Computer	Adults: *N* = 243; 59% female; age range = 18–60 years	France	FD	Yes	No	No	AFD	Yes	Yes	No
Raatz [39], 2015	Tap and Track OR Nutrihand	App, Smartphone OR Internet-based program, Computer	Adults; *N* = 19; 58% female; mean (SD) age = 51.6 (1.5) years	US	FD	No	No	No	AFD	No	Yes	No
Rangan [40], 2015	Electronic Dietary Intake Assessment (e-DIA)	App, Smartphone	Young adults; *N* = 80; 63% female; mean (SD) age = 21(0.5) years	AUS	FD, T	Yes	No	No	AFD, MCD	Yes	Yes	Yes
Rollo [43], 2015	Nutricam Dietary Assessment Method (NuDAM)	App, Smartphone	Adults (type 2 diabetes); *N* = 10; 40% female; mean (SD) age = 61.2 (6.9) years	AUS	I, V	Yes (In follow up phone call)	No	No	MCD	Yes	Yes	Yes
Svensson [44], 2015	Mobile phone app	App, Smartphone	Children; *N* = 81; 62% female; mean (SD) age = 15.5 (0.5) years	Sweden	FD, I	Yes	No	Yes	AFD	Yes	Yes	No
Timon [45], 2015	Novel Assessment of Nutrition and Ageing (NANA)	Internet-based program, Computer	Adults; *N* = 94; 63.8% female; mean age = 73 years	UK	FD, I, V	No	No	No	MCD	Yes	Yes	No
Delisle Nystrom [28], 2016	Tool for energy balance in children (TECH)	Camera and text message functions of a mobile phone	Children; *N* = 39; 44% female; mean (SD) age = 5.5 (0.5) years; Parents recorded intake	Sweden	I, T	No	No	No	MCD	No	Yes	No
Lassale [35], 2016	NutriNet-Sante Web-based 24-h dietary record	Internet-based program, Computer	Adults; *N* = 198; 48% female; mean (SD) age men = 50.2 (16.2) years Mean (SD) age female = 50.7 (16.8) years	France	FD	Yes	No	No	AFD	Yes	Yes	No
Rangan [41], 2016	Electronic Dietary Intake Assessment (e-DIA)	App, Smartphone	Young adults; *N* = 80; 63% female; mean (SD) age = 21(0.5) years	AUS	FD, T	Yes	No	No	AFD, MCD	Yes	Yes	Yes
Pendergast [38], 2017	FoodNow	App, Smartphone	Young adults; *N* = 90; 79% female; mean (SD) age = 24.9 (4.1) years	AUS	I, T, V	Yes	No	No	MCD	Yes	Yes	Yes

Dietary assessment methods are listed in chronological order

*App* application, *AUS* Australia, *BMI* Body mass index, *EMA* Ecological momentary assessment, *FR* food record, *GPS* geospacial positioning system, *N* sample size, *NR* not recorded, *PDA* personal digital assistant, *Prompt* Reminder to log food intake, *SD* standard deviation

Data entry Inputs: *I* image, *T* text description, *V* voice recording, *FD* select from food database, *B* barcode scanner

Coding method: *MCD* manual coding by dietitian, *AFD* automated from food database, *SAIA* semi-automatic image analysis

**Table 2:** Comparison of dietary intakes assessed using online or mobile food diaries with dietary intakes assessed using other dietary assessment methods or biomarkers of dietary intake and with total energy expenditure assessed using direct measures

First author (ref), year	Population	Test method and time frame^a^	Interval	Reference method and time frame^a^	Dietary intake variables	Correlation with EI or TEE; correlation range for other variables	Bland-Atman mean difference^b^ (95% limits of agreement) for EI	Comments
Comparison with direct measures of total energy expenditure								
Delisle -Nyström [28], 2016	Children; *N* = 39; 44% female; mean (SD) age = 5.5 (0.5) years old; parents recorded intake	Tool for Energy Balance in Children (TECH); 4 separate days of participants’ choice	0	DLW; 14 days	EI	NA	−220 kJ (−1760, 1320), *p* = 0.064 *Concerted to −52 kcal (−420,315), p = 0.064*	Mean difference for EI-TEE was non-significant. Mean change in bodyweight during test period was 0.07 ± 0.32 kg
Hutchesson [31], 2015	Young adults; *N* = 18; 100% female; mean (SD) age = 23.4 (2.9) years old^c^	Online FR; 7d (computer-based or smartphone-based with a 7-day washout period)	0	Indirect calorimetry (day 1) and accelerometry (7 days)	EI	NA	Computer-based: −510.2 kcal (−1288.9, 268.6); Smartphone-based: −455.7 kcal (−1290.9, 290.9)	Mean differences for EI-TEE were non-significant. Participants weight stable during test period.
Hutchesson [32], 2013	Adults; *N* = 9; 100% female; mean (SD) age = 34.5 (11.3) years old^c^	Online FR; 9 days	0	DLW, 10 days	EI	NA	−550 kcal (−1268, 168)	Pilot study.Energy (kJ/d) = −2301(1535) Participants weight stable during test period.
Kaczkowski [33], 2000	Adults; *N* = 53; 100% female; mean (SD) age = 64.9 (11.3) years old^c^	Multimedia Diet Record (MMDR); 4 days	0	two-point DLW, 13 days	EI	NA	NA	Mean difference in EI-TEE (−2.9 MJ) was significant (*P* < 0.01). Mean reporting accuracy was 76.0% (range 43–158%). Participants weight stable during test period.
Martin [36], 2012	Adults (BMI > 25 kg/m2); *N* = 40; 77.5% female; mean (SD) age = 40.3 (14.3) years old^c^	Remote Food Photography Method (RFPM) with customised or standard smartphone prompts; 6 days	0	DLW, 14 days	EI	NA	Standard prompts: −895 kcal (−2435, 645) Customised prompts: -270 kcal (−1766, 1226)	Pilot study BA plots showed no evidence of systematic bias Mean difference in EI -TEE was significant for the standard prompts (*P* < 0.001) only. Estimates of EI were adjusted for change in energy stores.
McClung [37], 2009	Young adults; *N* = 13; <1% female; Mean (SD) age = 23 (4) years old^c^	BalanceLog, 7 days	0	DLW, 7 days	EI	*r* = 0.60	−275 kcal (−1472, 920)	Mean difference in EI -TEE of 8% was not significant. Participants weight stable during test period.
Pendergast [38], 2017	Young adults; *N* = 90; 79% female; mean (SD) age = 24.9 (4.1) years old^c^	FoodNow; 4 non-consecutive days	0	Sensewear armband, 7 days	EI	*r* = 0.75 ICC = 0.75	−826.29 kJ (−3709.27, 2056.69) *Converted to -197 kcal(−886, 491)*	Analysis based on *n* = 56 after excluding energy misreporters. Participants weight stable during test period.
Rollo [43], 2015	Adults (type 2 diabetes); *N* = 10; 40% female; mean (SD) age = 61.2 (6.9) years old^c^	Nutricam Dietary Assessment Method (NuDAM); 3 non-consecutive days	0	DLW, 14 days	EI	NA	NA	Pilot study.Mean difference (EI-TEE) of 3 MJ was significant (*P* < 0.01). Mean EI:TEE ratio was 0.76.2 participants lost −2.8 kg in first week.
Svensson [44], 2015	Children; *N* = 81; 62% female; mean (SD) age = 15.5 (0.5) years old	Mobile phone app; 3 days	0	SenseWear Armband, 3 days	EI	*ρ* = 0.13 (*P* = 0.24)	-2586 kJ (−8285.6, 3113.68) *Converted to -610 kcal (−1980, 744)*	Assessed EI was 71% of TEE. BA plots showed 5 outliers. Not clear if participants were weight stable.
Comparison with other dietary assessment measures or dietary biomarkers								
Astell [24], 2014	Older Adults (BMI > 25 kg/m^2^); *N* = 40; 60% female; Mean (SD) age = 72.39 years old^c^	Novel Assessment of Nutrition and Ageing (NANA); 7 days	~6 weeks	4 day estimated FR (plus interview)	EI, protein, CHO, Fat	NA	−250 kJ (−1711, 1212) *Converted to* *-59 kcal (−409, 289)*	Mean difference in EI between methods was significant (*P* = 0.048). BA analysis also done for macronutrients; plots showed no evidence of bias.
Beasley [25], 2008	Adults (BMI > 25 kg/m^2^); *N* = 89; 83% female; Mean (SD) age = 52 (12) years old^c^	DietMatePro; 6 days (sampled across 4-w)	0	1 x 24HR	EI, protein, CHO, fat, SFA, cholesterol, fibre, vitamins A and C, calcium, iron	*ρ* = 0.542; *ρ* = 0.377 for vitamin C to *ρ* = 0.705 for cholesterol	NA	Results based on *n* = 71. Participants followed the Ornish prevention diet. Mean difference in EI between methods was not significant.
Beasley [26], 2005	Adults; *N* = 39; 54% female; mean (SD) age = 53 (1.7) years old	DietMatePro; 3 days	0	1 x 24HR	EI, protein, CHO, fat, SFA, cholesterol	*r* = 0.713; *r =* 0.505 for fat to *r =* 0.797 for CHO.	68 kcal (~ − 1600, 1600; limits not stated, approximation only)	Mean differences in dietary intakes between methods were not significant. BA Plot showed DietMatePro tended to overestimate EI relative to 24HR; 97% fell within ±2SD
Carter [27], 2013	Adults; *N* = 50; 72% female; mean (SD) age = 35 (9) years old^c^	My Meal Mate (MMM); 7 days	0	2 x 24HR- days chosen randomly	EI, protein, CHO, fat	*r* = 0.68; *r =* 0.57 for CHO *to r* = 0.75 for fat	206 kJ (−2434, 2022)*Converted to 49 kcal (−581, 483)*	Mean differences in dietary intakes between methods were not significant
Delisle Nyström [28], 2016	Children; *N* = 39; 44% female; mean (SD) age = 5.5 (0.5) years old; parents recorded intake	Tool for Energy Balance in Children (TECH); 4 separate days of participants’ choosing	0	4 x 24HR (with parent of child)	EI, fruits, vegetables, fruit juice, sweetened beverages, candy, ice cream, bakery products	*r =* 0.66; *ρ* = 0.665 for fruit juice to 0.896 for fruit and vegetables (combined)	NA	No significant differences for food group intakes (g) between methods.
Fukuo [29], 2009	Young adults; *N* = 44 without diabetes; 55% female; mean (SD) age = 23.2 (2.5) years old; *N* = 16 with diabetes; 19% female; mean (SD) age = 52.8 (9.9) years old^c^	PDA-based food diary; 1-day	0	1 x 24HR	Energy, protein, CHO, fat	Without diabetes: ICC = 0.854; ICC = 0.697 for CHO and 0.734 for fat. With diabetes: ICC = 0.801; ICC = 0.713 for protein to 0.796 for CHO	Done but results not shown	Mean differences in dietary intakes between methods were not significant. Authors stated that BA plots showed no evidence of bias
Higgins [30], 2009	Children; *N* = 28; 50% female; mean (SD) age = 12.6 (2.0) years old^c^	Photographic FR; 3 days (assessed by two separate dieticians)	0	3-day weighed metabolic diet	EI, Protein, CHO, fat, fibre, 6 micronutrients	*ρ* = 0.44 to 0.48; *ρ* = 0.06 for vitamin E to 0.80 for vitamin D	NA	ICC range = 0.25–0.92 for inter observer reliability (most ICCs >0.60). 50% subjects had missing photos.
Kikunaga [34], 2007	Adults; *N* = 75; 64% female; mean (SD) age = 48.8 (10.2) years old	Wellnavi method; 5 days	0	WFR, 5 days	EI, protein, fat, CHO, fibre, salt, cholesterol, 21 micronutrients	*ρ* = 0.602; *ρ* = 0.081 for Iron to 0.770 for vitamin B_12_	NA	Mean differences in EI and nutrients between methods was significant (*P* < 0.05) except for sodium, iron, vitamins A, D, E, K and B_12_, and cholesterol.
Lassale [35], 2016	Adults; *N* = 198; 48% female; mean (SD) age men = 50.2 (16.2) years old; mean (SD) age female = 50.7 (16.8) years old	NutriNet-Sante Web-based 24-h dietary record; 3 non-consecutive days over 2 weeks	<7 days before and <7 days after FR	Fasting blood concentration biomarkers (EPA, DHA, vitamin C, and β-carotene; collected at two separate visits ~3 weeks apart)	Vitamin C, beta-carotene, total n-3 PUFA, EPA, DHA, fruits and vegetables, fish and fatty fish	Men: *ρ* = 0.23 for n-3 PUFA to *ρ* = 0.58 for vitamin C; *ρ* = 0.20 for vegetables and plasma vitamin C to *ρ* = 0.55 for fish and plasma DHA Women: *ρ* = 0.32 for vitamin C to 0.38 for EPA; *ρ* = 0.13 for vegetables and plasma vitamin C to *ρ* = 0.55 for fish and plasma EPA + DHA	NA	Correlations were deattenuated and adjusted for age, weight status, smoking, education level, EI, alcohol, and supplements use.
Monnerie [49], 2015	Adults: *N* = 243; 59% female; age range = 18–60 years old	Estimated online FR; 7 days	1–2 weeks	Estimated FR, 7 days	EI, Protein, fat, CHO, simple CHO, alcohol, fibre, 8 micronutrients, 24 food groups, total water, total fluids, 8 beverage groups	NA	NA	Mean differences in intakes between methods for simple CHOs, Calcium, magnesium, vitamin D, 6 food groups, total water, total fluids and 4 beverage group were significant (*P* < 0.05)
Raatz [39], 2015	Adults; *N* = 19; 58% female; mean (SD) age = 51.6 (1.5) years old	Web-based dietary record (Nutrihand) or iPod-based Tap and Track program; 2 × 3 days	0	2 x Estimated FR, 3 days (entered by a dietitian)	EI, protein, fat, SFA, MUFA, PUFA, CHO, total sugars, fibre, cholesterol, 7 micronutrients	Nutrihand: *R* ^2^ = 0.56; *R* ^2^ = 0.02 for Vitamin A to *R* ^2^ = 0.88 for cholesterol. Tap and Track: *R* ^2^ = 0.01; *R* ^2^ = 0.00 for sodium to *R* ^2^ = 0.41 for total sugars	Nutrihand: 85.3 kcal (−851.5, 1022.1); Tap and Track: 100.6 kcal (−1748.7, 1547.5)	BA plots showed no evidence of systematic bias. Mean differences in dietary intakes between the methods were only significant for total sugars using Nutrihand (*P* < 0.05)
Rangan [40], 2015	Young adults; *N* = 80; 63% female; mean (SD) age = 21(0.5) years old^c^	Electronic Dietary Intake Assessment (e-DIA); 5 days	0	3 x 24HR, conducted on random days	EI, protein, fat, fat, SFA, MUFA, PUFA, CHO, total sugars, starch, cholesterol, fibre, alcohol, 14 micronutrients	*r =* 0.68; *r =* 0.55 for PUFA to *ρ* = 0.78 for Phosphorus	-34 kJ (−4062, 4130) *Converted to − 8.1 kcal (−970, 987)*	Vitamin and mineral supplements were excluded from the analysis. Correlations were deattenuated and energy; BA analysis also done for macronutrient intakes and plots showed no evidence of systematic bias. Mean differences in dietary intakes between were small and not significant
Rangan [41], 2016	Young adults; *N* = 80; 63% female; mean (SD) age = 21(0.5) years old^c^	Electronic Dietary Intake Assessment (e-DIA); 5 days	0	3 x 24HR, conducted on random days	Fruits, vegetables, grains, meat and alternatives, dairy and alternatives, discretionary foods, discretionary beverages, alcoholic beverages	*ρ* = 0.69 for discretionary beverages to *ρ* = 0.88 for discretionary food and for alcoholic beverages	Limits of agreement (e-DIA-24HR) ranged from −0.8 g (−124, 122) for meat and alternatives to 23.0 g (−293, 339) for discretionary beverages	BA plots showed no evidence of systematic bias. Median differences in food group intakes were not significant.
Rollo [43], 2015	Adults (type 2 diabetes); *N* = 10; 40% female; mean (SD) age = 61.2 (6.9) years old^c^	Nutricam Dietary Assessment Method (NuDAM); 3 non-consecutive days	<7 days	WFR, 3 non-consecutive days	EI, protein, fat, CHO, alcohol	*r* = 0.57; *r =* 0.24 for fat to *ρ* = 0.85 for alcohol	NA	Pilot study.Mean or median differences in dietary intakes were not significant.
Rollo [42], 2011	Adults (type 2 diabetes); *N* = 10; 40% female; mean (SD) age = 61.2 (6.9) years old^c^	Nutricam Dietary Assessment Method (NuDAM); 3 days	< 7 days	Estimated FR, 3 days	EI	NA	-649 kJ (−2269, 971) *Converted to -155 kcal (−542, 232)*	Feasibility study Mean difference in EI between methods was significant (*P* = 0.03).
Timon [45], 2015	Adults; *N* = 94; 63.8% female; mean age = 73 years oldTotal sample was derived from 3 separate studies	Novel Assessment of Nutrition and Ageing (NANA); 7 days	~6 weeks (FR); 1 week for blood draw	Estimated FR (plus interview), 4 days; fasting blood plasma ascorbic acid concentration, urine urea excretion	EI, protein, fat, SFA, CHO, NSP, Alcohol, 10 micronutrients	*r* = 0.879; *ρ* = 0.265 for vitamin B_12_ to *r* = 0.830 for CHO; *r* = 0.466 for protein with urine urea to *r* = 0.294 for vitamin C with plasma ascorbic acid	−249 kJ (−1887, 1389) *Converted to -59 kcal (−451, 332)*	Urinary analysis based on *n* = 76. Blood analysis based on *n* = 56. Mean differences in dietary intakes were significant (*P* < 0.05) for EI, protein, alcohol, vitamins A, B_6_, B_12_ and C, and carotenoids. *N* = 18 supplement users excluded from analyses
Touvier [46], 2011	Adults; *N* = 147; 59.2% female; mean (SD) age = 60.8 (6.0) years old^c^	NutriNet-Sante Web-based 24-h dietary record; 1 day	0	1 x 24HR (telephone)	Energy, protein, CHO, fat, SFA, MUFA, PUFA, cholesterol, fibre, ethanol, 20 micronutrients, 18 food groups	Men: *r* = 0.86; *r =* 0.68 for PUFA to 0.91 for vitamin D; ICC = 0.51 for fats and sauces to 0.92 for breakfast cereals. Women: *r =* 0.85; *r =* 0.56 for PUFA to *r =* 0.93 for fibre; ICC = 0.46 for cakes, biscuits and pastries to 0.94 for pulses	NA	Correlations adjusted for EIWeekend days were overrepresented
Wang [47], 2006	Young adults; *N* = 28;100% female: Mean (SD) age = 19.3 (0.5) years old^c^	Wellnavi method; 2 × 1 day (6 months apart	0	WFR, 2 × 1 day (6 months apart)	Energy, protein, fat, MUFA, PUFA CHO, cholesterol, fibre, salt, 22 micronutrients	*ρ* = 0.58 (June) and *ρ* = 0.60 (November). *ρ* = 0.21 for salt and sodium to *ρ* = 0.86 for vitamin K	NA	Median differences in EI between the methods at either time point were not significant. Median differences in nutrient intakes were significant (*P* < 0.05) for zinc, manganese, PUFA, fibre, SFA, vitamin E at one or both time points. Information on supplements also collected
Wang [48], 2002	Young adults; *N* = 20; 100% female; age NR (university students)^c^	Wellnavi method; 5 days	0	WFR, 5 days	Energy, protein, fat, MUFA, PUFA CHO, cholesterol, fibre, soluble fibre, insoluble fibre, salt, 23 micronutrients	*ρ* = 0.79; *ρ* = 0.38 for retinol to 0.93 for copper and vitamin B_12_	NA	Median difference of 6% in EI was not significant. Median differences for potassium, magnesium, copper, manganese, vitamins E, K and C, Folic acid and total fibre were significant (*P* < 0.05)

### Study Characteristics

The included studies were conducted in seven countries: seven studies were from Australia [31, 32, 38, 40–43], six from the USA [25, 26, 30, 36, 37, 39], four from Japan [29, 34, 47, 48], three from both the UK [24, 27, 45] and France [35, 46, 49], two from Sweden [28, 44] and one from Canada [33]. Studies were mostly conducted in females with 15 consisting of mostly female participants (>50%) [24–27, 29, 34, 36, 38–41, 44–46, 49] and five studies included female participants only [31–33, 47, 48]. Only five studies included mostly male participants (>50%) [28, 35, 37, 42, 43], and one study had even numbers of male and female participants [30]. The majority of studies were conducted on adults with 13 studies including adults of a wide age range (mean age 30–65 years) [25–27, 32–36, 39, 42, 43, 46, 49]. Seven studies were conducted on young adults (mean age 18–30 years) [29, 31, 37, 38, 40, 41, 47], and two studies were conducted on older adults (mean age > 65 years) [24, 45]. Only three studies were conducted on children (mean age < 18 years) [28, 30, 44]. One study did not report the age of its participants [48].

### Characteristics of the Dietary Assessment Method

Multiple platforms were used to administer food diary dietary assessment methods. The majority (*n* = 17) were administered via an application on a mobile phone or PDA handheld devices [25–27, 29, 31, 34, 36–44, 47, 48], while eight were administered via internet-based computer programs [24, 31, 32, 35, 39, 45, 46, 49]. One study used a camera alone [30], while another study used a combination of camera and microcassette recorder [33]. The final included study used the image and text functions present on mobile phones, whilw no specific program or application was required for this method [28].

Dietary assessment features ranged between methods. Data entry input methods included text descriptions, voice recording, images and selection from food databases. Of the 26 included studies, 15 used multiple data entry input methods [24, 27, 28, 33, 34, 38, 40–48], while nine used food database selection only [25, 26, 29, 31, 32, 35, 37, 39, 49] and two used images as their only form of data collection [30, 36].

Some methods incorporated prompts to encourage participants to remember to record their intakes with 11 of the 26 articles recording the use of EMA prompts. Delivery of these prompts varied between methods with smartphone or PDA methods utilising reminder text messages or pop up notifications throughout the day [25, 36, 38, 40, 41, 44], and reminders administered in follow-up phone calls the day following a reporting day [42, 43]. The internet-based programs on computers used multiple pass techniques frequently used in 24-h recalls to encourage accurate reporting during their eating occasion entries [35, 46, 49]. None of the included studies utilised global positioning services (GPS) to provide details on specific locations of consumption.

Data coding methods varied between food diaries with data entry input methods dictating coding method used. Studies that used food database selection input methods were able to use automated coding direct from the food item and amount selection from the database [25, 26, 29, 31, 32, 35, 37, 39, 44, 46, 49]. Of the 11 studies that used automated coding process, three studies provided instantaneous nutritional feedback to participants [31, 32, 44]. Twelve studies required manual coding of food and beverage data by trained nutrition staff or dietitians [24, 27, 28, 30, 33, 34, 38, 42, 43, 45, 47, 48], while three studies used a combination of automated and manual data checking processes [36, 40, 41].

Eating pattern assessment ranged across the 26 included studies. Only six included studies measured all three aspect of eating patterns (patterning, format and context) [38, 40–43, 46]. Format was the most commonly assessed out of the three aspects of dietary patterning with all 26 included studies assessing aspects of food combinations, nutrient content or sequencing of foods [24–49]. Patterning of intake including frequency, spacing, regularity, skipping or timing was reported by 15 of the included studies [24–27, 29, 35, 38, 40–46, 49], while context of eating occasions including eating with others location of eating or activity whilst eating was reported by six of the studies [38, 40–43, 46].

### Characteristics of the Food Diary Evaluations

#### Studies Using Direct Measures of Total Energy Expenditure as the Criterion Method

Nine of the included studies compared total energy intake with direct measures of total energy expenditure which was measured using the ‘gold standard’ DLW [28, 32, 33, 36, 37, 43], indirect calorimetry combined with accelerometry [31] or the SenseWear armband [38, 44]. For the test method, the number of days used to collect information on dietary intake ranged from 3 [28, 43] to 9 days [32]. Reference methods were usually conducted for a longer time period with the number of days for reference method collection ranging from 7 [37] and 14 days [28, 36] for DLW, 3 [44] and 7 days [38] for SenseWear and 7 days for accelerometry [31].

Among three of the nine studies that examined correlations [37, 38, 44], they ranged from 0.13 to 0.75 when compared to Sensewear, [44] [38] while the correlation was 0.60 for the only study using DLW [37]. Bland-Altman analysis in seven studies [28, 31, 32, 36–38, 44] showed that compared to DLW, mean differences (95% limits of agreement) in energy intake to energy expenditure ranged from −52 kcal (−420, 315) for the Tool for Energy Balance in Children (TECH) [28] to −895 kcal (−2435, 645) for the Remote Food Photograph Method (RMFM) [36]. The TECH and RFPM methods also reported the narrowest [28] and widest [36] 95% limits of agreement for estimated energy intakes to energy expenditure.

#### Studies Using Dietary Assessment Methods or Biomarkers as Reference Methods

Dietary intakes assessed using the test dietary intake method (e.g. online or mobile food diaries) were mostly compared to dietary intakes assessed using other dietary assessment methods as the reference method (19 studies) and included estimated [24, 39, 42, 45, 49] or weighed food records [34, 43, 47, 48] and 24-h recalls [25–29, 40, 41, 46] (Table 2). Two studies compared dietary intakes against blood or urine concentration biomarkers [35, 45], while one study used a weighed metabolic diet as a criterion reference method [30].

The dietary intake variables examined varied across studies with 11 studies [25, 30, 34, 35, 39, 40, 45–49] examining various micronutrients (usually in addition to total energy and macronutrient intake) and three studies examining various food groups [28, 35, 41, 49]. For the test methods, the number of days or time frame used to collect information on dietary intake ranged from 1 day [29, 46, 47] to 7 days [24, 27, 45, 49] with four studies reporting that they used non-consecutive dietary intake recording days [25, 28, 35, 43] and 11 incorporated at least one weekend day [24, 27, 35, 39–43, 45, 46, 49]. A similar number of dietary assessment days was usually adopted where estimated or weighed food records were used; however, the number of 24-h recalls varied from one [25, 26, 29, 46] to three or four non-consecutive days [40, 41].

Correlations for total energy intake ranged from 0.44 for 3-day photographic records compared with a 3-day weighed metabolic diet [30] to 0.88 for the 7-day Novel Assessment of Nutrition and Ageing (NANA) with 4-day estimated food records [45]. Most correlation coefficients for total energy intake were between 0.41 and 0.60 [25, 30, 39, 43, 47] or 0.61 and 0.80 [26, 28, 34, 40, 41, 48, 50], suggesting moderate or good correlations. Correlations for nutrient intakes were lowest (correlation range 0.0–0.4) for fat [43], sodium [39, 47], vitamin C [25], vitamin B_12_ [45] and retinol [48] and highest (e.g. correlations > 0.80) for cholesterol [39], alcohol [43], vitamin D [46], fibre [46], vitamin K [47], copper [47] and vitamin B_12_ [47]. All correlations for food groups were >0.4, with most correlations >0.60 [28, 41, 46]. Correlations for nutrients intakes or food groups with blood or urine biomarkers tended to be lower and ranged from 0.20 to 0.60 [35, 45]. Only three studies reported energy-adjusted correlations for nutrient intakes between methods [35, 40, 46], and only two studies deattenuated correlations for intra-individual variation in dietary intakes assessed using the reference method [35, 40]. Consideration of supplement intakes when analysing agreement or correlations for micronutrient intakes between methods was also rare [40, 45].

Eight [24, 26, 27, 39–42, 45] of the included studies conducted a Bland-Altman analysis (including plots) of the difference and limits of agreement for estimated intakes of energy and/or macronutrients/food groups between the test and reference methods. Mean differences (95% limits of agreement) in energy intake ranged from −8.1 kcal (−970, 987) for the Electronic Dietary Intake Assessement (e-DIA) compared with three 24-h recalls [41] to -155 kcal (−542, 232) for the Nutricam dietary assessment method (NuDAM) compared with an estimated food record in a feasibility study [42]. The mean difference with the narrowest 95% limits of agreement between methods was reported for the NANA (compared to an estimated food record; mean difference [95% limits of agreement] = −59 kcal [−409, 289]) [24], and the mean difference with the widest limits was reported for the Tap and Track method (compared to an estimated food record; mean difference [95% limits of agreement] = 101 kcal = [−179, 1548]) [39].

## Discussion

A recent scientific statement by the American Heart Association [4••] highlighted the significance of research in the area of eating patterns, and the need for research to address key gaps in this field has also been noted by the US Dietary Guidelines Scientific Advisory Board [9]. A core issue in progressing this research agenda relates to the need for dietary assessment methods that can capture the key constructs and variables of interest in relation to examining eating patterns that are acceptable to research participants and that can be used at the population level. This review focused on electronic and mobile food diary methods that have been developed and evaluated to assess food and beverage intake, and their capacity to be used to assess eating pattern exposures, including the patterning, format and context of eating occasions. Few studies reported the ability to assess all three of these domains, with eating occasion content and patterning the most common elements.

The electronic and mobile food diaries identified for this review included a range of technological elements for data entry and coding such as use of images, selection of foods from a database, text descriptions, automated coding and feedback to participants. Future developments should focus on expanding the range of components that could improve the reporting of eating occasions or decrease the burden for participants. For example, none of the identified tools reported use of barcode scanners or GPS capabilities. Barcode scanning is common among commercially available mobile phone apps developed for self-monitoring [23], although this requires linkage to commercial food product databases at the food brand level data [23]. GPS capabilities can allow assessment of location of eating and exposure to the food environment through mapping of location of eating and linkage to food environments data through Geographical Information Systems [51]. However, practical limitations have been noted relating to precision of the GPS assessments, difficulties in reliability accessing signals and impact on device battery power [51].

Currently, there is limited development of methods suitable for children and adolescents; however, there is promise in using these methods [52, 53] particularly where intake is assessed via the use of images only. While most studies identified in this review were conducted in adults, further work is required to assess their use among older adults who may be less familiar with online or mobile technologies and in participants with low literacy or low information and communication technology (ICT) literacy. However, web-based 24-h recall methods such as the Automated Self-Administered 24-Hour Recall (ASA24) have been shown to be acceptable even among older adults [54], which holds promise for other technology-based assessment tools. Previous reviews have identified the need for training of participants when using many of these tools [14] and it maybe that these aspects require further development for specialist populations, although research suggests that some tools may be feasible in diverse community groups [55].

Validity of each of the electronic or mobile food diaries was evaluated using a range of reference methods including comparisons to other food diaries (weighed and estimated), 24-h recalls, DLW and SenseWear measure of energy expenditure and plasma and urine biomarkers. Validity of the methods was found to be similar to traditional dietary assessment methods, consistent with other reviews of technology-based dietary assessment methods [56]. Currently, all studies focused on validating total dietary intake which most directly relates to eating pattern format variables (e.g. energy and nutrient content and distribution across eating occasions). However, none of the existing studies evaluated the ability to assess other elements of eating pattern constructs such as timing and frequency of eating occasions and factors relating to context of eating. Alternative approaches to validation such as the use of wearable cameras may provide future opportunities to objectively asses and validate eating pattern context factors such as location and presence of other people [22, 57].

This review was restricted to dietary assessment methods where an evaluation or validation study had been published. Therefore, other existing methods were excluded, where no published validation study is currently available [58–60], including some commercially developed tools designed for self-monitoring rather than for research [61] or where the validation study was published after completion of this search [62]. This review also focused on methods that allowed estimation of total food and nutrient intakes and thus excluded other novel technologies such as wearable cameras [63–65] and bite counters [66] whose application to dietary assessment is currently limited to providing supplementary data to augment dietary data collection, but do not currently capture total food and beverage intakes. The field of technology-based dietary assessment is rapidly developing and new methods may soon be evaluated or become available that allow assessment of eating pattern factors, and therefore, researchers must critically evaluate each new method, in relation to the advantages and disadvantages before selecting a tool [67, 68].

While having a number of potential advantages, electronic and mobile food diaries and records may still exhibit a number of the same limitations as traditional (pen and paper) methods [13••, 14]. For example, regardless of the mode of administration, food diaries may result in reactivity, that is, the act of recording may lead people to change their food and beverage intake due to the burden and participants may be susceptible to social desirability bias [14]. Study design considerations for traditional methods are also equally relevant, for example the use of multiple days to account for day-to-day variation and intake of occasionally consumed foods, inclusion of weekday and weekend days and the use of non-consecutive reporting days to account for correlated intakes [69, 70].

An advantage of the use of electronic or mobile assessment techniques is the potential to incorporate measures of a wide range of factors in food diaries that have not previously been examined concurrently with food intake in population-based studies. Electronic and mobile methods lend themselves to these assessments due to the ability to rapidly collect large amounts of real-time data. For example, alongside food and beverage intake at each eating occasion, factors relating to ingestive behaviours such as appetite and satiety [71] and potential outcomes, including mood-related factors, fatigue and alertness can be assessed [72–74]. Eating pattern assessment can be conducted concurrently with the use of other ambulatory monitoring techniques and wearable devices to assess other health behaviours such as physical activity, sedentary behaviour and sleep using pedometers and accelerometers and health outcomes such as blood glucose via continuous glucose meters [75]. These data may be considered ‘microlongitudinal’ in nature and can be used to examine time-lagged effects and bidirectional relationships, relating to the next meal and those on subsequent days [76]. Future research relating to eating patterns will require the development and application of appropriate statistical techniques, as the data collected on eating occasions is hierarchical in nature, as the repeated assessments of eating occasions are nested within individuals [75]. Furthermore, identification of statistical techniques that can simultaneously analyse the patterning, format and context of eating occasions over time is needed to better understand the complexity of everyday eating situations.

## Conclusions

Eating patterns have been identified as an international research priority area. Technology-based dietary assessment method and, specifically, electronic and mobile food diary methods provide significant opportunities to expand this area of research and address key research gaps. To date, a diverse range of methods are available for examining the patterning and format/content of eating occasions, but tools that address the contextual aspects of eating patterns are more limited. Further consideration of eating pattern constructs is required when developing dietary assessment methods.
